# Not all heroes wear capes: a photovoice exploration of youth experiences during and beyond COVID-19

**DOI:** 10.1186/s12889-026-26731-8

**Published:** 2026-02-20

**Authors:** Varalakshmi Chandra Sekaran, Akshaya S Chandrasekaran, Ajith K Remesan, Nikitha Sibil Rebello, Monali Bhattacharya, Lena Ashok, Brayal Carry Dsouza, Naveen Kumar

**Affiliations:** 1 Department of Global Public Health Policy and Governance, Prasanna School of Public Health, Manipal Academy of Higher Education, Manipal, India; 2 Department of Social and Health Innovation, Prasanna School of Public Health, Manipal Academy of Higher Education, Manipal, India; 3 Department of Healthcare and Hospital Management, Prasanna School of Public Health, Manipal Academy of Higher Education, Manipal, India; 4 RAK College of Medical Sciences, RAK Medical and Health Science University, Ras Al Khaimah, UAE

**Keywords:** COVID-19, Youth Mental Health, Young adults, Lived experiences, Photovoice

## Abstract

**Background:**

The COVID-19 pandemic prompted significant transitions in many lives, with UNICEF reporting that 73% sought help for physical and mental well-being. This study is grounded in life course theory, examining youths’ emotional and behavioral adaptations during this period. The study was carried out to explore the lived experiences of youth during and after the COVID-19 pandemic through photovoice methodology.

**Methods:**

Employing a qualitative, community-based participatory approach, the study was conducted among students aged 18 to 25 years. Data collection in this study is conducted using photovoice methodology, a participant-led research approach in which the researcher and the participant collaborate through photographs and narratives. Data were gathered from participants in Udupi, Karnataka, through 72 photographs and 24 journals, complemented by one-on-one interviews using the SHOWeD prompt technique.

**Results:**

Twenty-four youths (8 males, 16 females) from India, Malaysia, and Sri Lanka participated. Four primary themes emerged: fears leading to resilience, societal pressures, coping strategies during adversity, and new beginnings post-pandemic. Youth utilized various mechanisms, including social support and spirituality.

**Conclusion:**

The study highlights youth’s adaptive strategies and resilience amid significant challenges, utilizing the photovoice approach.

**Supplementary Information:**

The online version contains supplementary material available at 10.1186/s12889-026-26731-8.

## Background

On March 11, 2020, the World Health Organization (WHO) officially declared COVID-19 as a global pandemic and the pandemic took a toll on people’s lives in ways that have never been seen before [1]. Alongside the physical burden, many other consequences, such as confinement-related psychological anguish and social distancing measures, influenced everyone; different age groups were affected differently [2, 3]. Recent studies have shown that youth groups were more prone to psychological problems due to the COVID-19 pandemic [4, 5].

Young individuals were limited in their contact with friends, relatives, family, and many others due to social distancing guidelines increasing their loneliness and subjecting them to mental health problems [6, 7]. It was also seen that among various challenges such as psychological distress, anxiety, stress, and post-traumatic stress disorder posed upon youth in general, the pandemic’s unprecedented nature disrupted traditional education, transitioning into online education [8, 9]. This rapid switch from in-person to online learning made students adjust to new living circumstances, which might have added stressors among students [10, 11].

Specifically, healthcare professional students whose courses are designed to interact with patients and have hands-on experiences seem to have a clear disadvantage to them [12, 13]. Apart from providing education, the traditional education environment buffers youth against the negative consequences of stressors, including social interaction, physical exercise, consistent means, and a structured routine, which were disrupted due to the COVID-19 pandemic [14]. These abrupt disruptions for prolonged periods and other pandemic-related stressors have been found to increase the risk for psychological distress among youth [15, 16].

In Sri Lanka, the mental well-being of youth was affected due to COVID-19 [17], and a study found that during the COVID-19 pandemic, depressive symptoms was reported by 40.8% young adults, while anxiety was reported by 34%, and elevated stress level was found among 24.7% young adults [18]. In addition, depression (12.5%), anxiety (15.2%), and stress symptoms (6.4%) were prevalent among young adults in Malaysia during the COVID-19 pandemic [19]. However, an Indian study found that 87% of the Indian youth perceived moderate to a high levels of psychological stress during the COVID-19 pandemic [20].

The Ecological Systems Theory by Urie Bronfenbrenner emphasizes the interconnection between developmental trajectories and significant transition points, which can include impactful sociohistorical events like the COVID-19 pandemic [21]. This theoretical framework provides a nuanced perspective for exploring the layered and interconnected environmental systems that influence development. The microsystem represents the most immediate environment, including family, peers, and school [22]. The mesosystem encompasses the interactions between different microsystems [23]. The exosystem consists of systems that indirectly affect the individual, such as financial stability, college environment, and parental workplace [24]. The macrosystem includes laws, beliefs, cultural values, and societal norms; in this case, it pertains to pandemic-related news, lockdown measures, societal attitudes towards masks, and vaccines [25]. Lastly, the chronosystem considers the dimension of time and how the pandemic influences individuals over different periods [26]. Applying the theory enables researchers to systematically unpack the complex interplay of factors that shaped the experiences of youth during the pandemic.

In the study, the photovoice methodology provides a unique glimpse into the lived experiences of youth surrounding the COVID-19 pandemic. The photovoice approach is a type of research driven by participants, where researchers and participants collaborate by using photographs and personal stories. The photovoice method used in this research involved participants taking photographs reflecting their daily lives. Participants were also asked to keep a journal throughout specific days. Afterwards, individual interviews were held, using the photographs as visual cues during the conversations [27–29].

Limited studies have explored the lived experiences through the lens of healthcare professional students during the COVID-19 pandemic. This study aims to explore the lived experiences of youth during both phases of COVID-19 and in the post-COVID period using the photovoice methodology.

## Methods

### Participants

The institutional ethics committee of the tertiary care hospital approved the study (IEC1:17/2022). This study was conducted among undergraduate students from a university campus in Udupi taluk, Karnataka. A taluk is an administrative area that is further divided into cities, towns, or villages. The inclusion criteria for the study were young adults aged above 18 years pursuing undergraduate degrees in a university campus in India which included young adults from Indian and international backgrounds, who were willing to participate in the study. To explore the lived experiences of young adults from different countries and those who are from medical and non-medical background, potential participants from this age group were identified by purposive sampling.

Within the period of the study, a total of 25 students were invited to participate, including a mix of medical and health science students, of whom 24 consented to participate in the study, and one withdrew. All the students belonged to a university campus in India and of all 24 students who participated, 8 were male, and 16 were female (Table 1).

**Table 1:** Participant profile

Participant names	Age	Gender	Course	Nationality
P1	21	F	Medicine	Malaysia
P2	22	F	Medicine	Sri Lanka
P3	22	F	Medicine	Sri Lanka
P4	21	M	Medicine	Malaysia
P5	23	F	Medicine	Malaysia
P6	22	M	Medicine	Malaysia
P7	23	F	Medicine	Malaysia
P8	21	F	Medicine	Sri Lanka
P9	22	F	Medicine	Malaysia
P10	22	M	Medicine	Sri Lanka
P11	23	F	Medicine	Sri Lanka
P12	21	F	Medicine	Sri Lanka
P13	22	M	Medicine	Malaysia
P14	22	F	Medicine	Sri Lanka
P13	22	F	Social Work	India
P14	22	F	Social Work	India
P15	22	F	Social Work	India
P16	20	F	Social Work	India
P17	21	F	Social Work	India
P18	21	M	Social Work	India
P19	22	M	Social Work	India
P20	22	F	Social Work	India
P21	24	M	Social Work	India
P22	26	M	Social Work	India

### Data collection

Data collection in this study was conducted from May 2022 to April 2023 using the photovoice methodology, a participant-led research approach in which the researcher and the participant collaborate through photographs and narratives [27, 28]. The photovoice methodology used in this study captured the thoughts and emotions or any event of the participant’s interest to understand their lived experience in the post-COVID setting.

The study was conducted as follows: During the initial meeting with the participants in their comfortable space, the study’s objective was explained, and they were asked to capture their thoughts and emotions by submitting photographs and maintaining a journal for 10 days. An in-depth interview was scheduled with the participant after agreeing on a convenient date, place, and time. During the interview, the participant’s submitted photographs and journals served as tools for them to share their lived experiences of different phases of COVID-19.

This approach allowed the participant to express their own voice, providing rich and profound insights [30]. The assignment involved capturing the photograph and journaling their experience related to their experience in a post-COVID setting that happened during the 10 days, after which the photography release forms were obtained. A total of 72 photographs and 24 journals were collected, following which indepth interviews (IDIs) were conducted in English language as per participant’s preference.

### In-depth interviewing

As it was a participant-led approach, along with the in-depth interview guide (see Additional file 1: IDI Guide), the narratives were initiated through photographs and journal entries submitted by the participants. However, the following broad themes were probed to gain insights in keeping with research objectives: experiences during COVID-19, coping during COVID-19, family relationships during COVID-19, and new normal experiences post-COVID-19. These themes were examined through the lens of the meanings associated with the photographs and journal entries. Each interview lasted for a duration of 45 to 60 min, and the SHOWeD technique [31, 32] was used as a prompt that consisted of different questions that relate to the photograph, such as: What do you see here? What is really happening here? How does this relate to our lives? Why does this problem, concern, or strength exist? What can we do about it?

The interviews were audio recorded, and notes were taken simultaneously; later, it was transcribed into English and checked for accuracy. The study spanned the data collected between May 2022 to April 2023, and the data collected were anonymized and accessed only by the research team. The researcher provided counselling and was, where necessary, by a social work professional to help participants work through feelings that may have arisen during the interviews.

### Data processing and analysis

The transcripts were fed into ATLAS. Ti, version 8. The analytical method involved a single compiled data file for each participant, including visual data from photographs, journal content, and interview data. Later, the compiled data file was used to develop codes, sub-themes, and themes as they emerged into narratives. The themes were both deductive and inductive [33].

## Results

After a comprehensive thematic analysis of the data collected, the following significant themes emerged (Table 2). The results are arranged in the sequence of the following themes: Fears leading to resilience, the shift in approaches to learning, coping during COVID-19, and new beginnings in the post-COVID period.

**Table 2:** Themes and sub-themes

1. Fears leading to resilience
-New norms surrounding COVID-19
-Facing fears and showing resilience
2. The shift in approaches to learning
-Closure of educational institutions
-Learning in the midst of home and family responsibilities
3. Coping during COVID-19
-Engaging in shared activities with family
-Shouldering responsibilities
4. New beginnings in the post-COVID period
-Hope in the face of adversity
-Mixed feelings in the immediate post-COVID period

### Theme 3.1: fears leading to resilience

The pandemic was a new experience to everyone and the fear of pandemic along with the new norms, staying away from daily routines and close ones seemed to be difficult in the initial stages. Getting adapted to the new routines was a challenge for the participants.

#### Sub theme: 3.1.1 new norms surrounding COVID-19

Participants, through their lived experiences, shared that changing routines during the COVID-19 pandemic resulted in experiences surrounding lockdowns, quarantine, isolation, and the establishment of new norms such as masks, sanitizers, and oxygen saturation monitoring becoming part of routine life.

**Fig. 1:**
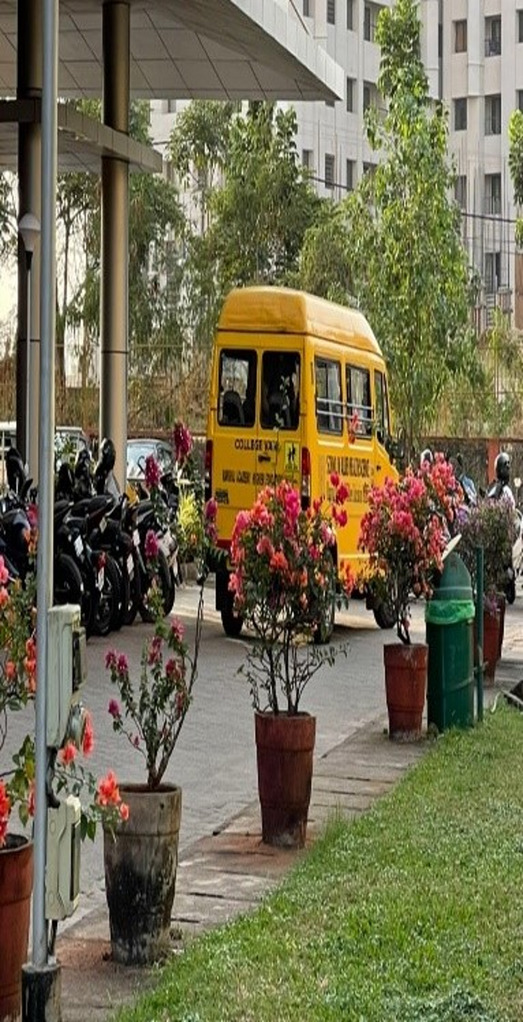
Van to pick up COVID-19 patient for isolation (P4, male)

**Fig. 2:**
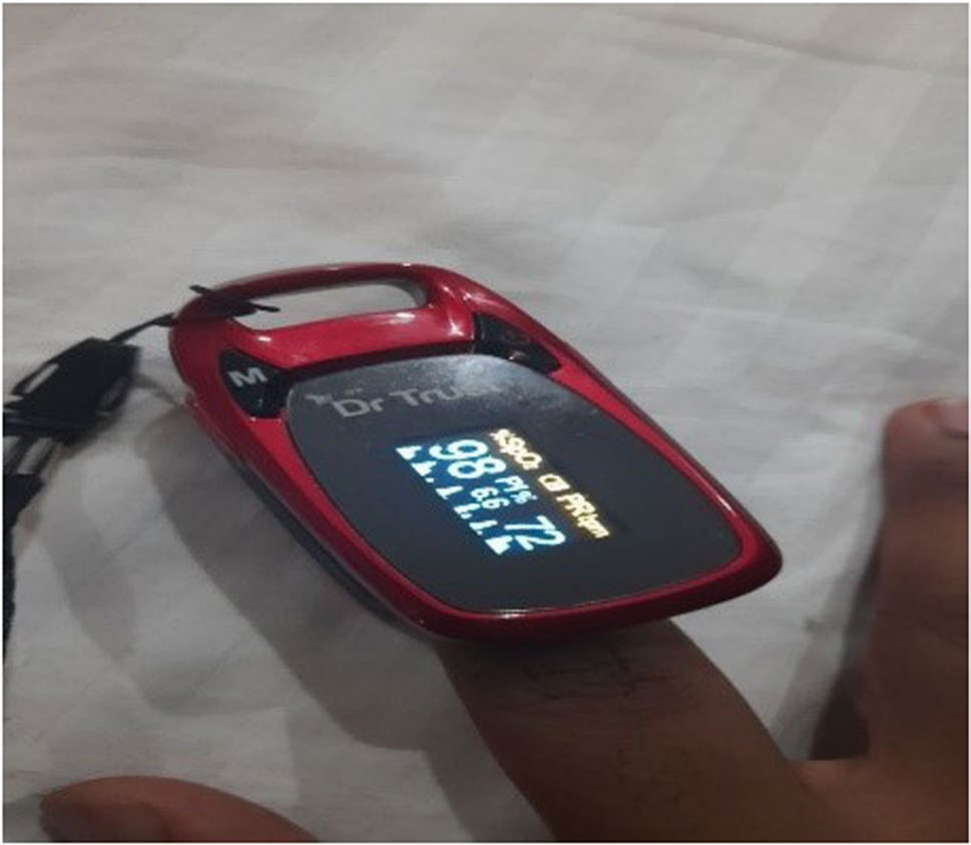
‘New essentials to have’ (P21, male)

“Masks were made mandatory; random COVID testing was done; RT-PCR became mandatory when traveling overseas; people carried sanitizers everywhere and at the back of their minds were still anxious about physical contact. Temperature checks were done before entering places… and slowly but surely, this became the new norm.” (Journal entry, P3, female) (Figs. 1 and 2).

#### Sub-theme: 3.1.1 facing fears and showing resilience

**Fig. 3:**
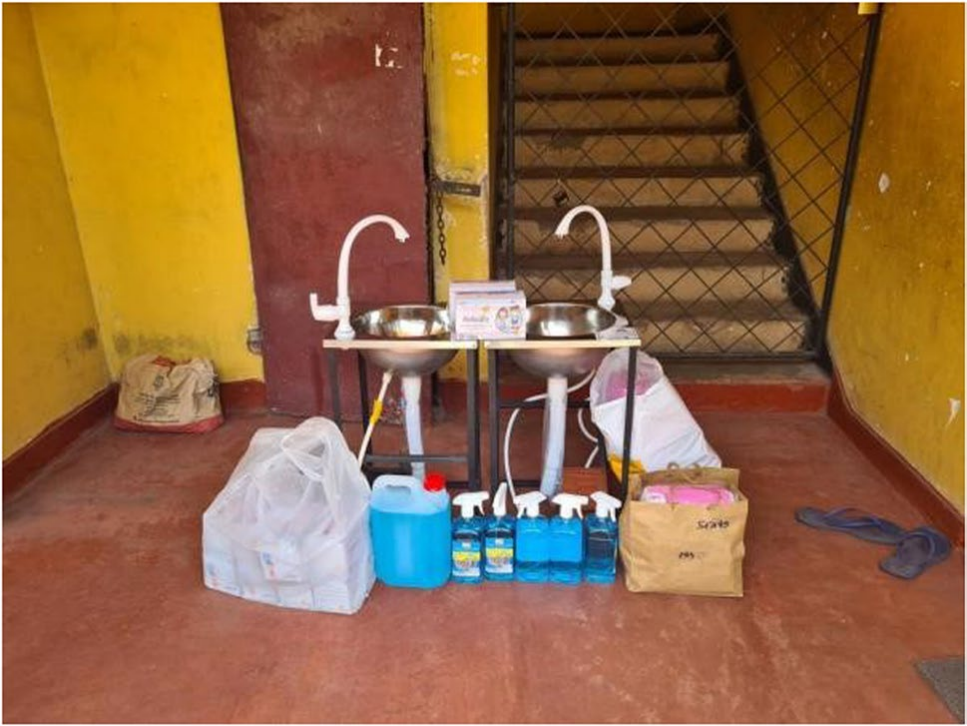
Some people also donated things like hand wash, wash basins, hand sanitizers, etc., to places where they were needed but not afforded. (P14, female)

**Fig. 4:**
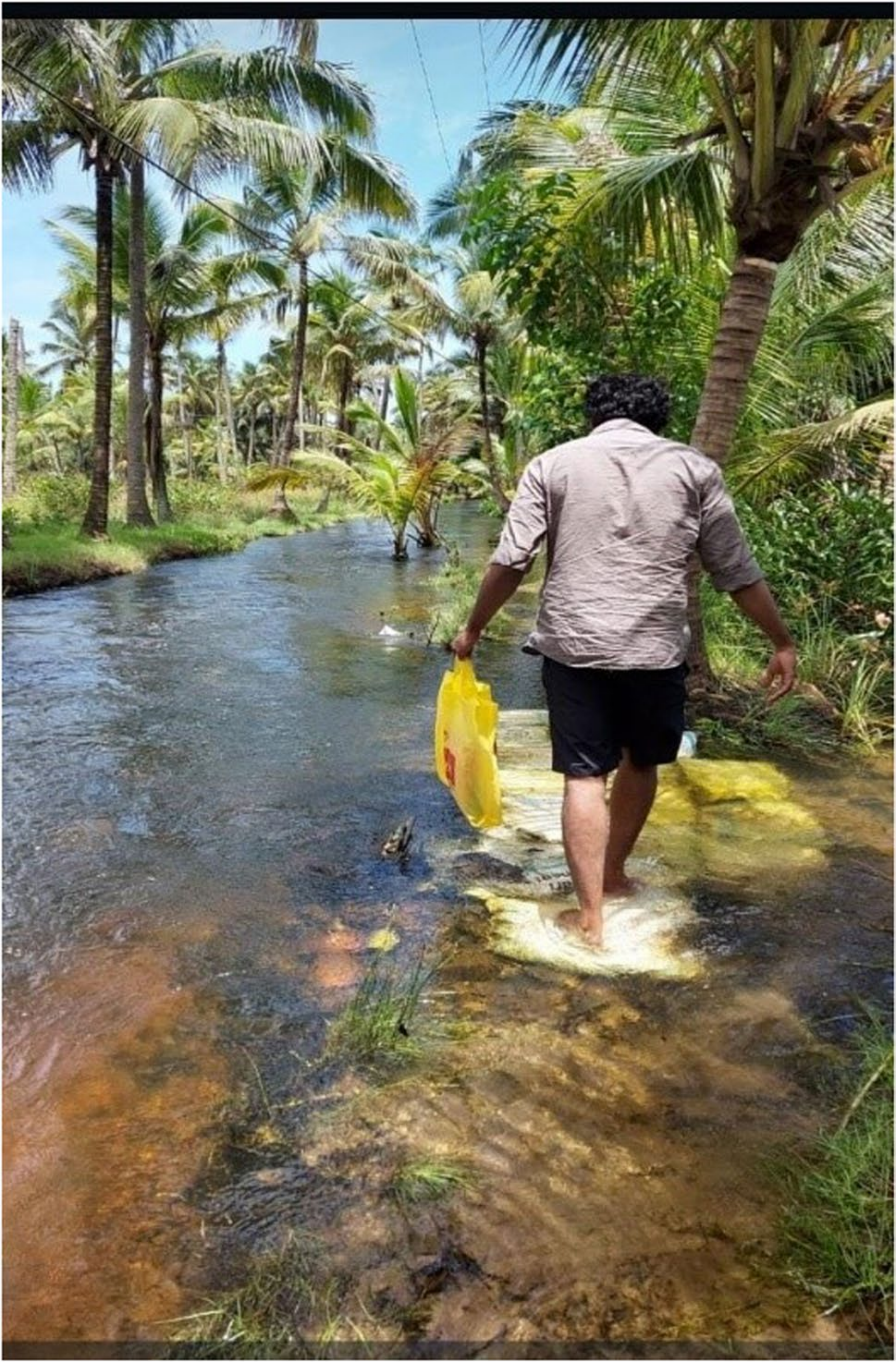
Going to a quarantine house to give supplies (P22, male)

**Fig. 5:**
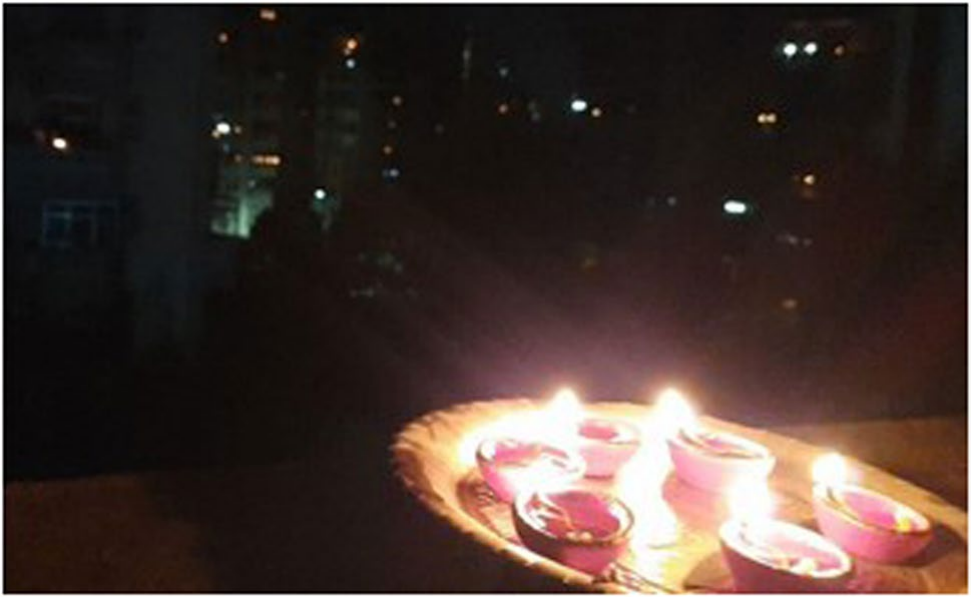
Lighting diyas on my balcony to show solidarity with frontline workers (P21, male)

Enduring difficult circumstances, navigating through rising numbers of cases and hospitalizations as well as facing the reality of mortality around them became the new norm. Despite the various fears that troubled the youth during the pandemic, the shared experience of living through a pandemic also showed glimpses of resilience and adaptiveness to the new norm. Expressing their solidarity also brought altruistic behaviors to the fore. These found expression in the form of support to frontline workers combating the pandemic, donating essentials to those in need, and providing home supplies to families during quarantine (Figs. 3, 4 and 5).

“Others may view those videos of doctors crying and dying as scary or terrifying, but it just made my dream to be a doctor firmer. To me, they were like one of those heroes working in the background together as a team. As people always say, “Not all heroes wear capes.” (Journal entry, P1, female).

### Theme 3.2: shift in approaches to learning

The COVID-19 pandemic significantly impacted the education system, prompting a shift from traditional methods. Educational institutions were compelled to implement alternative approaches to cope with the new circumstances, presenting challenges for everyone involved.

#### Sub-theme: 3.2.1: closure of educational institutions

**Fig. 6:**
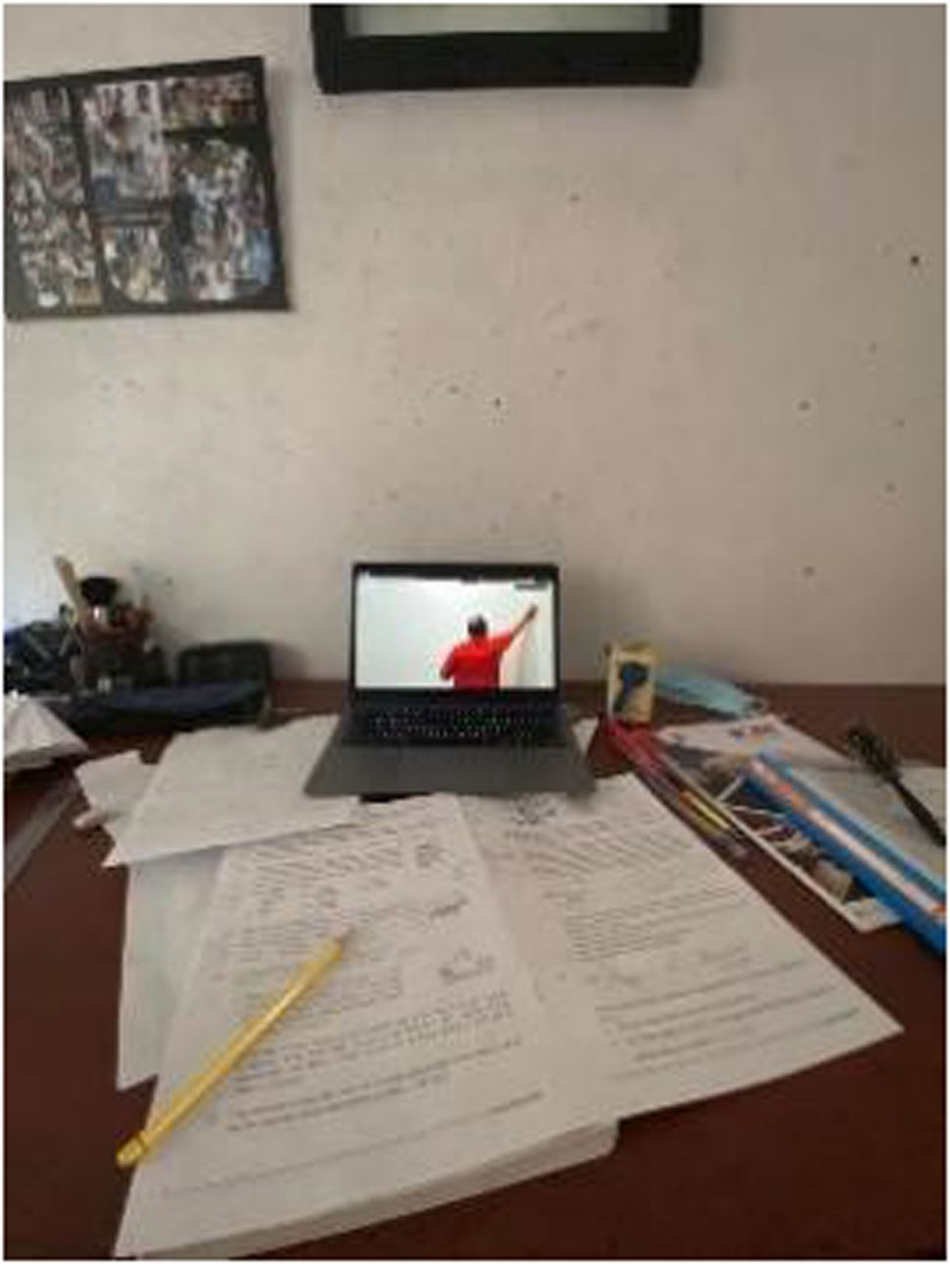
‘Classes were shifted online’ (P14, female)

One of the significant and dramatic shifts students were exposed to included changes in the learning environment and the mode of delivery of learning content. The shift from traditional in-person learning to online learning placed pressure upon students, especially those pursuing careers in healthcare. Participants expressed their and their siblings’ lived experiences during this transition as following statements:

“Sitting in my room in front of my laptop from 8 am to 3.30 pm sometimes never gave me the feeling that I was studying medicine… one virus has taken in all our lives.” (Journal entry, P12, Female) (Fig. 6).

“I had online classes for almost a year. During this period, I was very confused and extremely distracted, and as we had to have even anatomy dissections online, it was very tiring.” (Journal entry, P 12, female).

#### Sub-theme 3.2.2: learning in the midst of home and family responsibilities

Learning in home spaces also proved particularly challenging as they had to make.

non-traditional spaces into learning spaces.

“Due to renovation and construction noises, I could not attend online classes and study peacefully during that period… beginning of the first year MBBS course online was really challenging. I even came to the point of giving up the course.” (Journal entry, P4, Male).

“I felt anxious as the college kept delaying our schedule… when the online classes started, I had a hard time trying to cope with the amount of material being taught.” (Journal entry, P6, Male).

### Theme 3.3: coping during COVID-19

Family dynamics played a central role in the coping strategies of young adults. With regular routines disrupted and social interaction essentially shifting online, many young people turned to their families for support, connection, and shared activities. However, there were participants who couldn’t choose family as a support system.

#### Sub-theme 3.3.1: engaging is shared activities with family

Most participants identified their parents, relatives, and peers as their primary coping support. While contact with peers was largely online, engaging in shared activities and having shared meals was an eagerly anticipated activity that families engaged in. The traditional practice of sitting together for a meal in the South Asian context was often repeated.

“Being at home with everyone all days for me was a rewarding experience. It strengthened our bonds with quality time with our loved ones, such times are truly blessed and gifted.” (Journal entry, P12, female).

“I realized that families who share everyday activities together form strong emotional ties.” (Journal entry, P4, male).

“I spent a lot of time with my mother and helped her cook and ended up learning a bunch of new recipes. We also did some gardening and ended up growing a bunch of vegetables and fruits.” (Journal entry, P8, female).

#### Sub-theme 3.3.2: shouldering responsibilities

However, some participants had additional family responsibilities, which left them drained with the concerns of the ongoing pandemic and having to manage online learning. One female participant had to care for her younger siblings and mother, who had unfortunately been affected by a stroke.

“Whenever I had an exam, I would be stressed because I had to study and take care of my mom at night since she would wake up and ask me to change her diapers. I was very tired mentally and physically. At these stressful times, I always wished that it would be very nice if I was in the hostel just studying.” (Journal entry, P2, female).

**Fig. 7:**
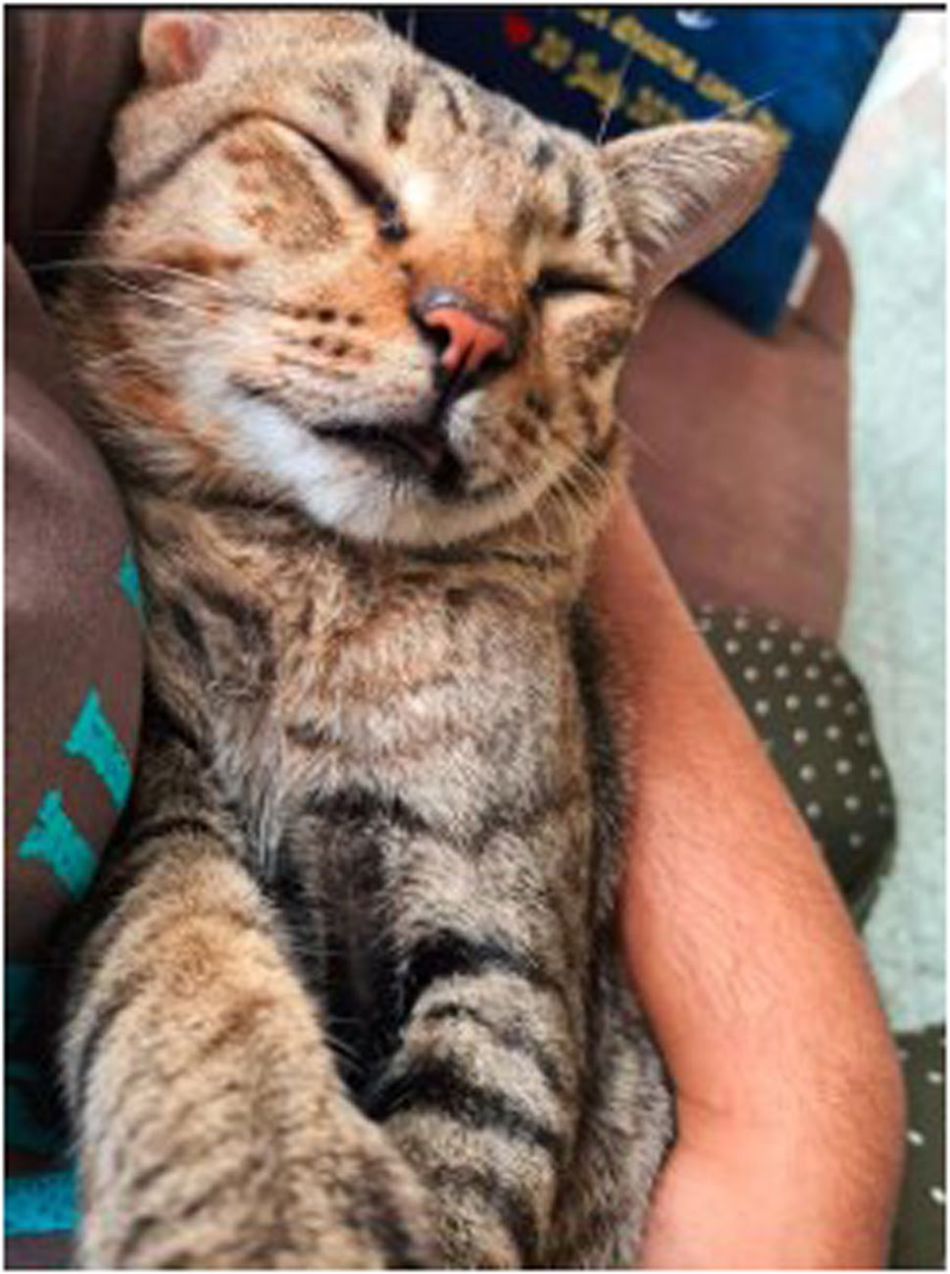
My cat ‘Kiki’ (P2, female)

She shared a picture of her pet cat ‘Kiki’ who had become her confidant. She stated,

“For me, although my house was filled with my family members and relatives, I still felt that I could not share my problems with anyone there. The only person that I could share my personal issues is my mother, but she is a stroke patient who can only listen for a short period of time what I talk about, she cannot respond to me. It has been 5 years since this happened, and it affected me a lot during lockdown because I had a lot of time with my family and got reminded of that.” She coped with the difficulties by growing close to her cat: “I was just happy that someone was there for me.” (Journal entry, P2, Female) (Fig. 7).

**Fig. 8:**
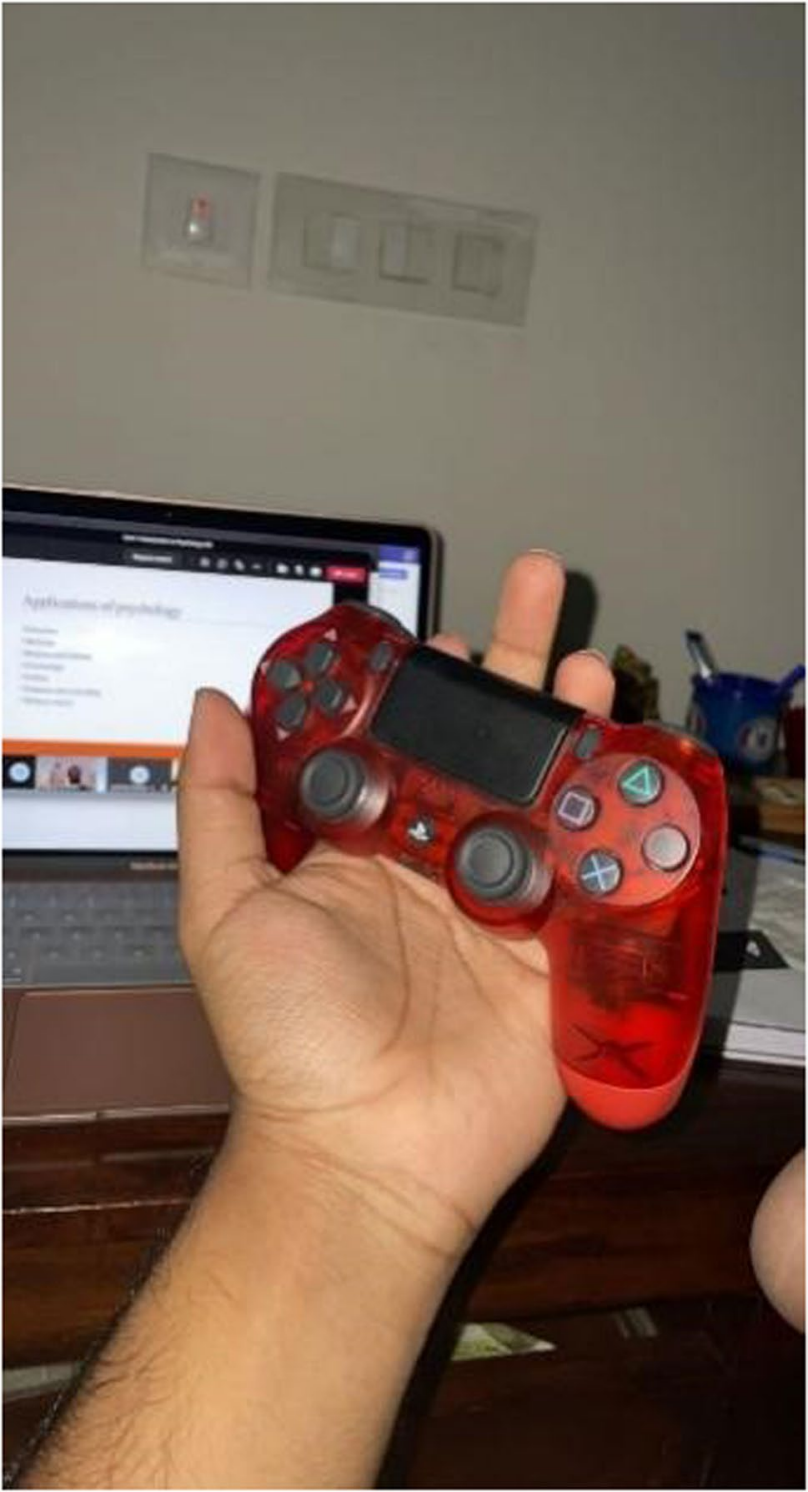
‘Laptop and PS4 are two things that got me through lockdown’ (P 22, male)

While some of them had to shoulder significant responsibilities, other youth engaged in learning new skills, such as trading in the online market or immersing themselves in self-help literature. Online activity and extensive gaming were common activities that youth engaged in (Fig. 8). 

### Theme 3.4: new beginnings in the post-COVID period

In the post-COVID period, young adults expressed a sense of renewal and hope as they resumed academic and career pursuits. At the same time, vaccinations marked a step toward normalcy, transitioning back to offline life brought mixed emotions, ranging from excitement and determination to lingering anxieties and reflections on the profound impact of the pandemic experience.

#### Sub theme 3.4.1: hope in the face of adversity

**Fig. 9:**
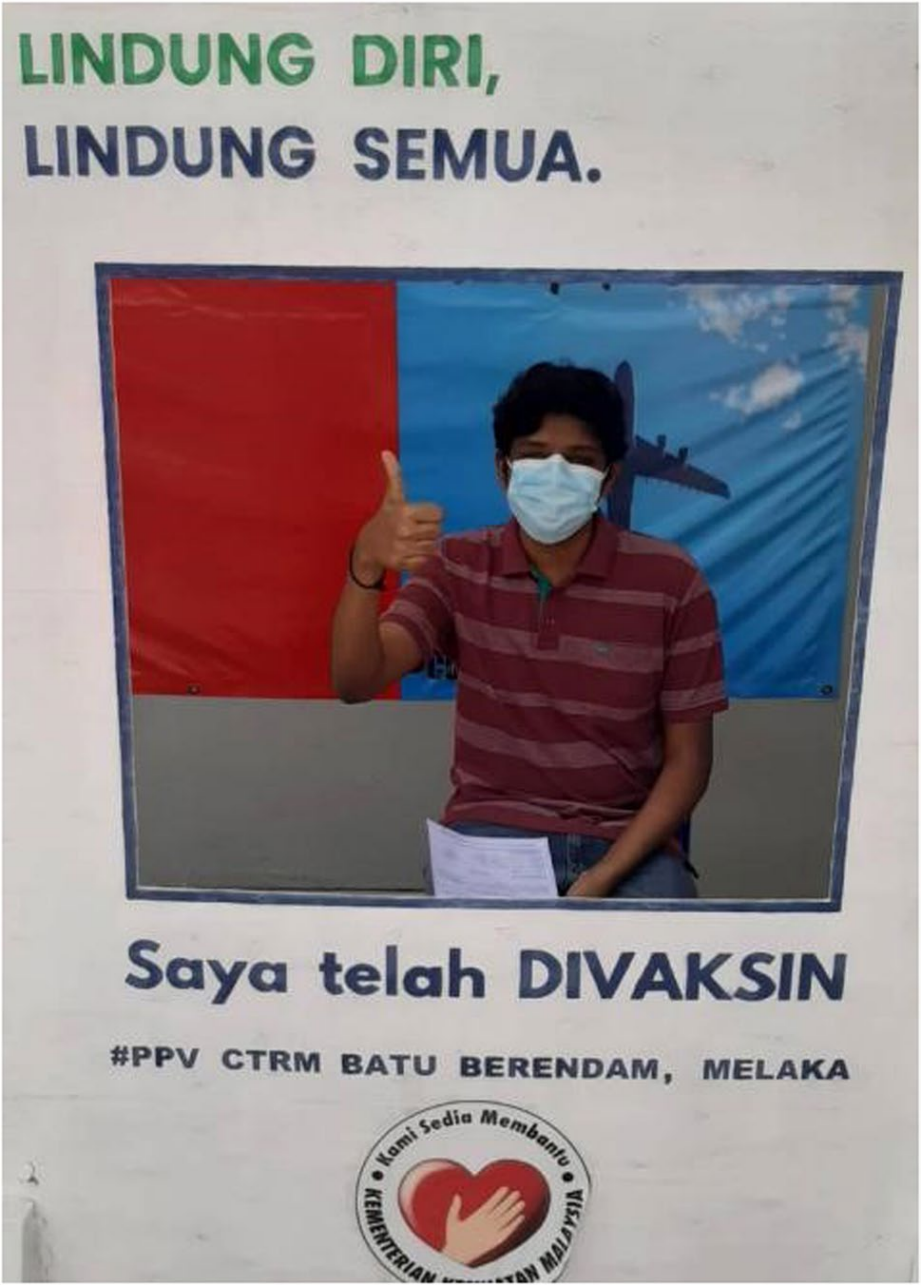
‘Vaccination done’ (P4, male)

Moving beyond these experiences, the young participants looked forward to moving beyond the COVID experience, having received the vaccination and anticipated returning to routines that would set them on the chosen career path. One participant reminisced on his thoughts immediately following vaccination (Fig. 9):

Leaving home to chase our dreams to build the path to a career that I always wanted to do gave me a lot of feelings.” (Journal entry, P12, female).

**Fig. 10:**
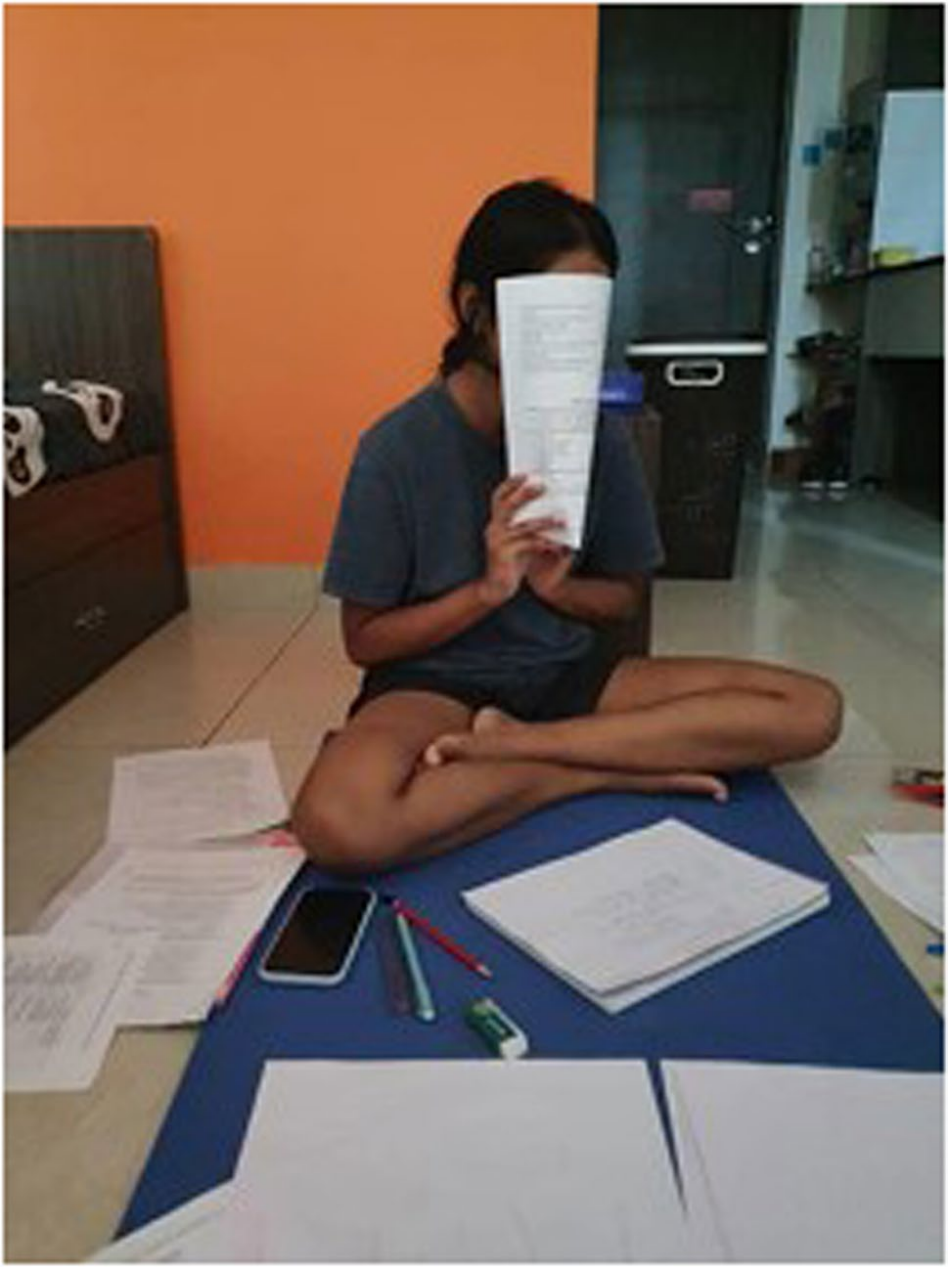
Offline life after an online life (P12, female)

Adjusting back to newer learning challenges continued even after the pandemic. A female participant expressed the after-effects of online learning:

“Compared to what happened around the world- people losing jobs, people losing their families- what we as students went through is quite small. Yet, from a college student’s perspective, the anxiety from moving to an offline life after an online life was real; adjusting took time and effort.” (Journal entry, P12, female) (Fig. 10).

#### Sub-theme 3.4.2: mixed feelings in the immediate post-COVID period

**Fig. 11:**
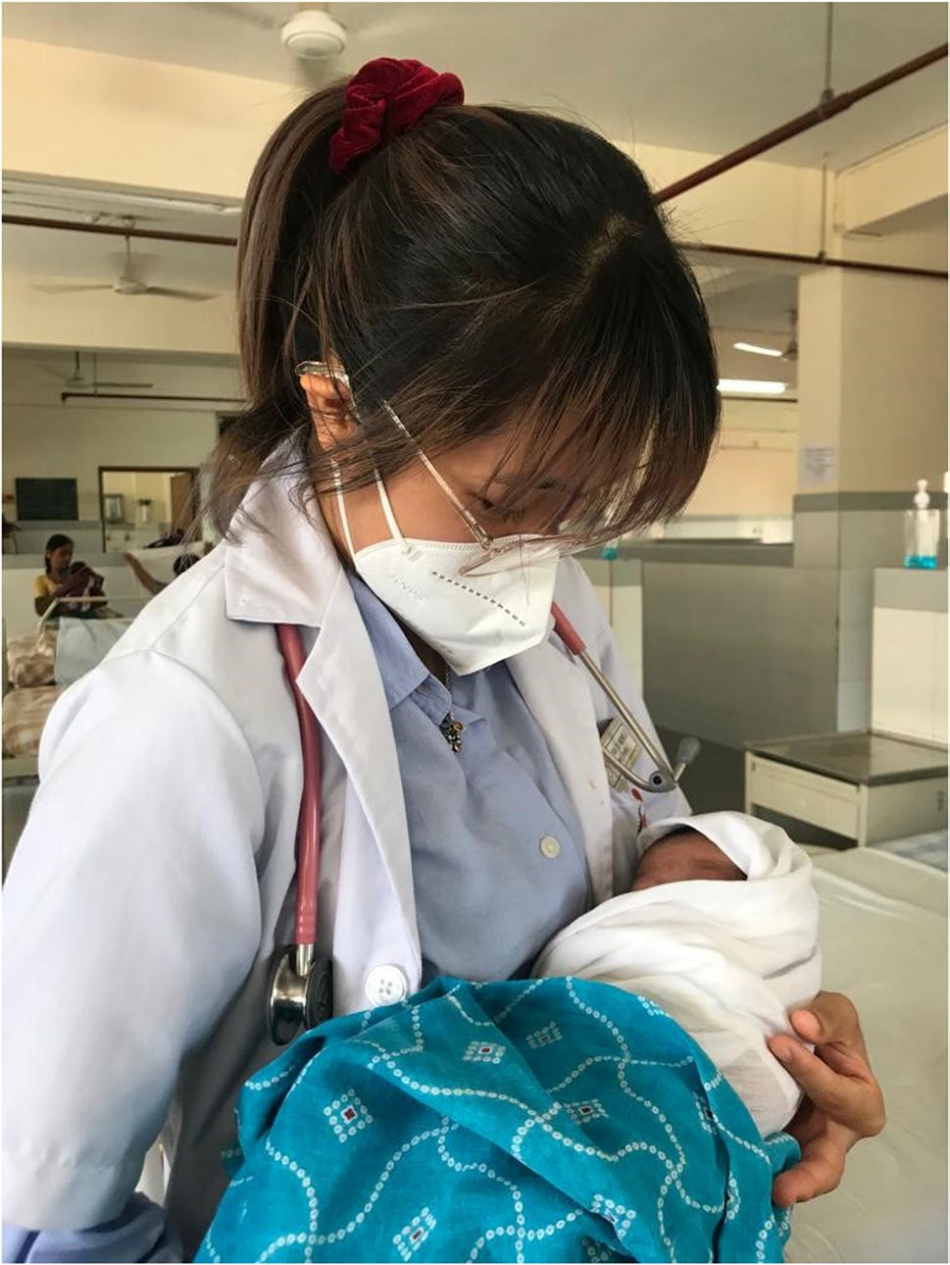
Wearing masks to protect others (P9, female)

**Fig. 12:**
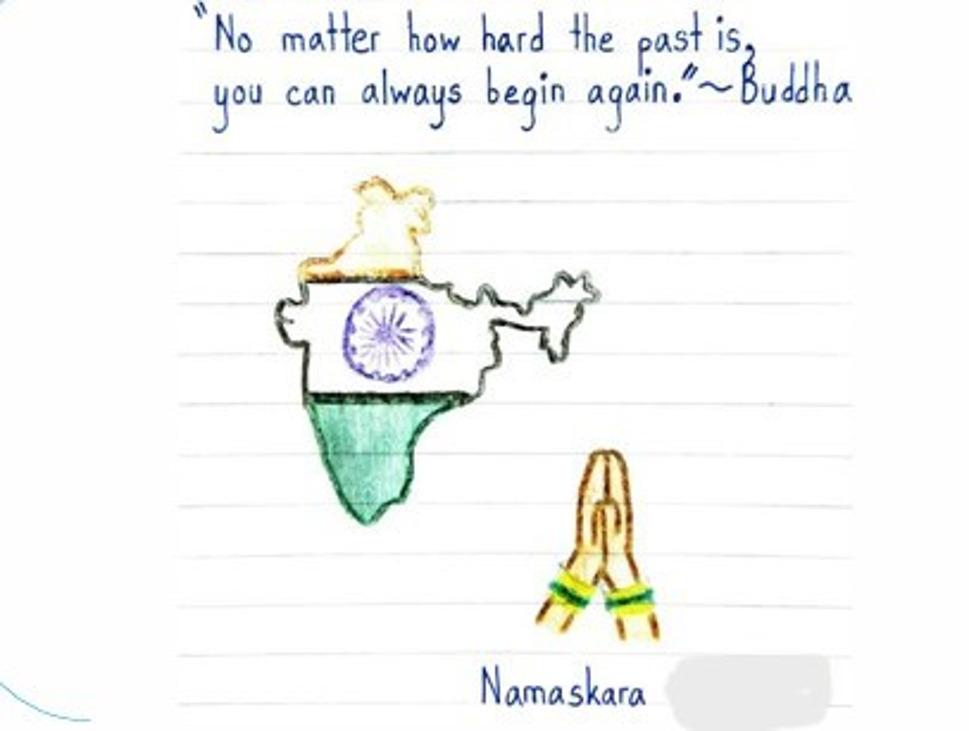
Begin again (Journal entry, P4, male)

Despite the new normal in the immediate post-COVID period, thoughts surrounding the pandemic experience gave rise to insights learned from the experience. Participants expressed mixed feelings once the lockdown was lifted, as they returned to their regular routines and resumed classes on the Indian campus. One participant referred to the pandemic experience as missing the ‘simpler times’ despite the immense once-in-a-lifetime experience of having lived through the pandemic:

“After getting vaccinated, after 1.5 years of online learning, I finally got the golden opportunity to travel to India, resuming offline classes… I felt extremely heartbroken to leave home… it was a mixed feeling for me… I knew that I needed to fulfill my dream as a successful doctor and bring honor to myself and my family.” (Journal entry, P4, male) (Figs. 11and 12).

## Discussion and conclusion

Our study explored the lived experiences of youth during different phases of COVID-19 and in the post-COVID period using the photovoice methodology bringing rare insights. The SHOWeD prompts used during interviews encouraged the participants to critically examine their photos and how specific experiences shaped their perceptions. The lived experiences of youth during different phases of COVID-19 revealed a compelling story of resilience, adaptability, and hope. By using photovoice methodology, researchers gained a deeper understanding of the complexities of youth experiences [30].

The pandemic’s sudden onset and rapid spread instilled fear in youths, especially regarding missing the opportunity to experience a traditional educational environment, socialization, health, education, and many other prospects. Despite the increase in COVID-19 cases, the death rate of Sri Lanka was at 0.48% which was considerably lower than the global rate of 2.14% [34]. In addition, between March 2020 and May 2021, Malaysia had reported 571,901 cases and 2,796 deaths with a case fatality rates (CFR) of 0.6% [35]. In India, the overall CFR was 1.16 per 1000 person-days, which declined from 1.80 per 1000 person-days during the first wave to 0.77 per 1000 person-days in the second wave. The risk of death was 1.49 times higher during the first wave and 35% lower in the second wave [36].

According to literature, the prevalence of depression, anxiety, and stress significantly increased among young adults during this period [37, 38]. This study found that such fears were also forerunners to resilient actions, as youths utilized their personal strengths and social networks to navigate through uncertain situations. For example, participants reported finding comfort in simple acts of solidarity, such as lighting diyas to support the frontline workers, donating essentials to those in need, and providing home supplies to families in quarantine. Such symbolic actions fostered a sense of shared strength, reminding youth that they were not alone in their struggles.

The pandemic heightened existing societal pressures, especially in education and fears about the future and careers [39]. Studies observed that crisis disrupted routines and introduced psychological distress, which required young adults to cope and adapt [40]. Participants in this study shared their lived experiences of transitioning from online classes back to in-person exams, which also posed significant academic and emotional challenges. Similarly, international participants underscore the effort required to reengage with traditional learning after prolonged remote learning.

Research indicates that youth face numerous difficulties with digital learning, including the non-conducive learning environment, internet connectivity issues, and psychological stress due to academic pressures [41]. Despite these challenges, the post-pandemic period offered opportunities for growth, and youth reported a renewed sense of purpose in their academic pursuits by engaging in self-directed learning and leveraging digital study tools to maintain their academic performance.

This research shows that young people primarily sought emotional support from family and peers, and their quality relationship directly impacted their mental health outcomes. This aligns with Bronfenbrenner’s ecological system theory, which emphasizes how interconnected systems influence an individual’s behavior and well-being [21]. During the pandemic, the microsystem- comprising family, friends, and immediate social circle- played an important role in the youth’s coping during the pandemic. A systematic review found that support from parents and positive peer relationships were key external protective factors that helped youth maintain resilience [42].

Studies have also found that to avoid the detrimental effect of social isolation, it is crucial to maintain social support systems such as friends and peer circles to maintain positive mental health during the pandemic [43, 44]. Other than quality time with friends, family, and relatives, this research has highlighted varied coping strategies among participants, reflecting cultural and individual differences. For instance, youths found new interests and habits to explore that kept their boredom routine at bay, such as gardening, cooking, baking, reading books, gaming, and spiritualism. The photovoice methodology allowed participants to document these coping mechanisms, reflecting a rich narrative of resilience.

Though loss and uncertainty were marked by the COVID-19 pandemic, it also catalyzed new beginnings. Participants expressed a sense of gratitude, for the pandemic was not merely a period of survival but also a time of reflection and transformation. Participants expressed their development of personal strength, appreciation of life, and deeper relationships resonating with post-traumatic growth. Globally, the pandemic has brought up hitherto unheard of difficulties that have an impact on people individually, in families, in communities, and society. Youth were able to utilize commonplace skills, connections, and resources to safeguard their mental health. The lesson learned during this unprecedented crisis is that even during adversity, youths have the strength and hope to chart new paths forward.

## Limitations

This study did not gain a deeper understanding of the intersectionality of other factors that influence the experiences and coping strategies, such as age, gender, cultural difference, socioeconomic status, and family background. It also did not explore the lived experiences of participants from non-health backgrounds; hence, lacking generalizability. These gaps need addressing and can be considered as the way forward to understanding the comprehensive lived experience of youths during adversities. In addition, future research during the pandemics where there are limitations for one on one interaction, the method of online photovoice also can be utilised [45, 46].

## Supplementary Information

Supplementary Material 1.

## Data Availability

Data is provided within the manuscript.
