# Supplementary material for: Not all heroes wear capes: a photovoice exploration of youth experiences during and beyond COVID-19
Source: BMC Public Health. 2026 Feb 20;26:747. doi: 10.1186/s12889-026-26731-8 (PMC12937526; doi:10.1186/s12889-026-26731-8)
Supplement: Supplementary file 1 — Supplementary Material 1. [file 12889_2026_26731_MOESM1_ESM.pdf]

Not all heroes wear capes: A photovoice exploration of youth experiences during and beyond COVID-19

IDI Guide

1. **Key questions: Using the SHOWed prompt for the photographs:**
    a. How this picture is related to you?
    b. Is there any particular reason for sharing this?
    c. What is your emotions about this?
    d. How you connect this picture with your life?
2. **Introduction**
    a. Can you please introduce yourself (name, age, where you're from)?
    b. What about your family?
    c. Briefly explain what you study and your interests.
3. **Life before the pandemic**
    a. What was your daily routine like before the COVID-19 pandemic?
    b. How would you describe your social life and support network then?
4. **Experience during the pandemic**
    a. How did you first hear about COVID-19?
    b. What were your immediate thoughts or feelings when the pandemic was announced?
5. **Daily life changes**
    a. How did your daily routine change once lockdowns began?
    b. What activities or routines did you miss the most during this time?
    c. How the shift to online education affected you?
6. **Emotional responses**
    a. What emotions dominated your experience during the pandemic (e.g., fear, sadness, anxiety)?
    b. Did you experience any changes in your mental health?
7. **Fears and resilience**
    a. What fears did you encounter during the pandemic?
    b. Can you describe a moment where you felt remarkably resilient?
8. **Societal pressures**

    a. Did you feel any pressure or expectations from society or peers during this time?
    b. How did these pressures affect your behaviour or feelings?

9. **Coping strategies**
    a. What coping strategies did you use to manage stress during the pandemic?
    b. Did you seek social support from friends or family? How did that help?
    c. Were any activities (e.g., hobbies, meditation) that helped you cope?
10. **Additional supports**
    a. Did you turn to any resources (online therapy, community programs) for help?
    b. How adequate were these resources in supporting you?
11. **New beginnings post-pandemic**
    a. As restrictions ease, what changes do you notice in your life?
    b. What new habits or routines have you adopted since the pandemic began?
    c. How has your perspective on life or goals changed post-pandemic?
12. **Reflection and conclusion**
    a. What were the most valuable lessons you learned during the pandemic?
    b. Do you feel better prepared for future challenges after your experience?
13. **Future outlook**
    a. What are your aspirations or goals moving forward?
    b. How do you envision your life a year from now?
14. **Final thoughts**
    a. Is there anything else you want to share about your experiences during COVID-19?
    b. How do you hope your story will impact others who are listening?
